# An anteromedial stabilization procedure has the most protective effect on the anterior cruciate ligament in tibial external rotation. A human knee model study

**DOI:** 10.1007/s00402-024-05357-8

**Published:** 2024-05-10

**Authors:** Fabian Blanke, Matthias Boljen, Nicola Oehler, Christoph Lutter, Thomas Tischer, Stephan Vogt

**Affiliations:** 1 Department of Knee-, Shoulder- and Hip-Surgery and Orthopedic Sports Medicine, Schön Klinik München Harlaching, München, Germany; 2 Department of Orthopedic Surgery, University Rostock, Rostock, Germany; 3 Fraunhofer Institute for High-Speed Dynamics, Ernst-Mach-Institut, EMI, Freiburg i, Breisgau, Germany; 4 Department of Orthopedic Sports Medicine and Arthroscopic Surgery, Hessing Stiftung Augsburg, Augsburg, Germany

**Keywords:** AML, Anteromedial stabilization, ACL rupture, ALL

## Abstract

**Introduction:**

Anterior cruciate ligament (ACL) reconstruction remains associated with the risk of re-rupture and persisting rotational instability. Additional extraarticular anterolateral stabilization procedures stabilize the tibial internal rotation and lead to lower ACL failure rate and improved knee stability. However, data for additional stabilization of tibial external rotation is lacking and the importance of an anteromedial stabilization procedure is less well evaluated. Aim of this study is to investigate the influence of an extraarticular anteromedial stabilization procedure for the stabilization of the tibial external rotation and protection of the ACL from these rotational forces.

**Methods:**

Internal and external rotations of the tibia were applied to a finite element (FE) model with anatomical ACL, posterior cruciate ligament (PCL), lateral collateral ligament (LCL), medial collateral ligament (MCL) and intact medial and lateral meniscus. Five additional anatomic structures (Anteromedial stabilization/anteromedial ligament, *AML*, augmented superficial medial collateral ligament, *sMCL*, posterior oblique ligament, *POL*, anterolateral ligament, *ALL*, and popliteal tendon, *PLT*) were added to the FE model separately and then combined. The force histories within all structures were measured and determined for each case.

**Results:**

The anteromedial stabilization or imaginary AML was the main secondary stabilizer of tibial external rotation (90% of overall ACL force reduction). The AML reduced the load on the ACL by 9% in tibial external rotation which could not be achieved by an augmented sMCL (-1%). The AML had no influence in tibial internal rotation (-1%). In the combined measurements with all additional structures (AML, ALL, PLT, POL) the load on the ACL was reduced by 10% in tibial external rotation.

**Conclusion:**

This study showed that an additional anteromedial stabilization procedure secures the tibial external rotation and has the most protective effect on the ACL during these external rotational forces.

**Supplementary Information:**

The online version contains supplementary material available at 10.1007/s00402-024-05357-8.

## Introduction

Anterior cruciate ligament (ACL) reconstruction still has a risk of re-rupture and persisting rotational instability and outcomes after ACL revision surgery are worse [1–4]. It is proven that an additional extraarticular anterolateral stabilization procedure or reconstruction of the anterolateral ligament (ALL) can lead to lower ACL reconstruction failure rate and improved knee stability [5–10]. Due to its oblique course the ALL stabilizes besides anterior translation the internal rotation of the tibia and therefore secures the ACL graft when high forces acting on the knee joint. However, the ALL is not able to stabilize the external rotation of the tibia which often is part of the ACL injury mechanism, especially in combination with valgus stressing, a fact that is often overlooked [11–14]. Therefore, to stabilize the tibial external rotation anteromedial structures should be brought into focus [15]. The fact of high rates of concomitant MCL ruptures during ACL injuries confirms this assumption [16, 17]. Therefore, medial structures should be addressed to restore anteromedial knee stability after an ACL injury and to protect the ACL graft after reconstruction [18–23]. To stabilize the knee and the ACL graft in external tibial rotation, a medial structure should be obviously constituted like a mirrored ALL from the lateral side. It should extend obliquely from the medial femoral epicondyle to the anterior medial part of the proximal tibia. This is already backed up by increasing interest in the medial side in ACL injuries and the importance of the complex architecture of the whole MCL with influence of the different fibers like the more anterior superficial MCL (sMCL) and the posterior oblique ligament (POL) [15–17, 23–25].

Thus, the primary objective of the present study was to assess the efficacy of different parts of the MCL, including an extraarticular anteromedial stabilization procedure, in enhancing knee stability, particularly in tibial external rotation. We evaluated this in a finite element knee model study to assess the impact on the rotational stability of the knee joint and on the ACL itself. Accordingly to the ALL on the lateral side the corresponding structure targeted for reconstruction on the medial side in the anteromedial stabilization procedure was termed the *anteromedial ligament*. We hypothesized that an extraarticular anteromedial stabilization procedure could serve as the primary stabilizer against tibial external rotation and offer superior protection to the ACL against these rotational forces compared to other medial or lateral extraarticular structures.

## Materials

### Human knee model

The Global Human Body Model Consortium (GHBMC) full human body model represents a 50th percentile male in an upright standing posture. The model has been developed by GHBMC [26] and is commonly used for traffic accident simulations involving human beings as occupants and pedestrians in combination with the explicit finite element solver LS-DYNA (Livermore Software Technology Corporation, LSTC) [27]. The knee angle has been adjusted to 25° in order to acquire a more critical reference configuration for ACL ruptures. In this configuration, the reference lengths of the ligaments were stated in a recent publication [15]. All material properties and contact definitions in the model components were kept constant and have not been modified.

The same material model with distinct properties for tension and compression (*MAT_PLASTICITY_COMPRESSION_TENSION) already used for the existing knee ligaments has been assigned to the additional extra-articular structures. The material model used for the ligaments follows an approach by Untaroiu et al. [28]. Since ligaments are much stiffer in tension than in compression, an isotropic elastic-plastic material model with different properties in compression and tension is used. Material parameters were described in an earlier publication [15]. Since identical material parameters are applied to all ligaments (including AML), the numerical simulation can quickly reveal a relative load distribution. The predominantly loaded ligaments can be identified easily using this approach, as long as the applied moment is not excessively high and the stress level in the ligaments is not near failure. The average tensile stress-strain curve reported by Quapp et al. [29] and a less stiffer curve were assigned to the material model in tension and compression, respectively [30]. The isolated FE model (knee joint) has been cut approximatively 170 mm above and 140 mm below the tibia plateau. The exact cutting edges are along the given spatial discretization. The model consists of roughly 50,000 elements and 37.000 nodes. All physical components (bones, muscles, ligaments, soft tissue) are organized by a total of 32 components. The isolated knee model encompasses a volume of 3.7 L and has a mass of 4.2 kg. The nodes on both cutting surfaces, on the femoral side and on the tibia side, are kinematically constrained so that no relative movement of the nodes is possible. While the rigid section of the tibia is fixed, the rigid section of the femur is loaded instantaneously by a constant moment of 20 Nm in order to establish both scenarios, femoral external and femoral internal rotation. The duration of the simulation was 100 ms. Since the final nodal positions are almost constant after approximately 60 ms, the final rotation angle and the normal forces within the internal ligaments and the extra-articular structures were averaged from this point of time to the end of the simulation.

### Measurement

**Fig. 1:**
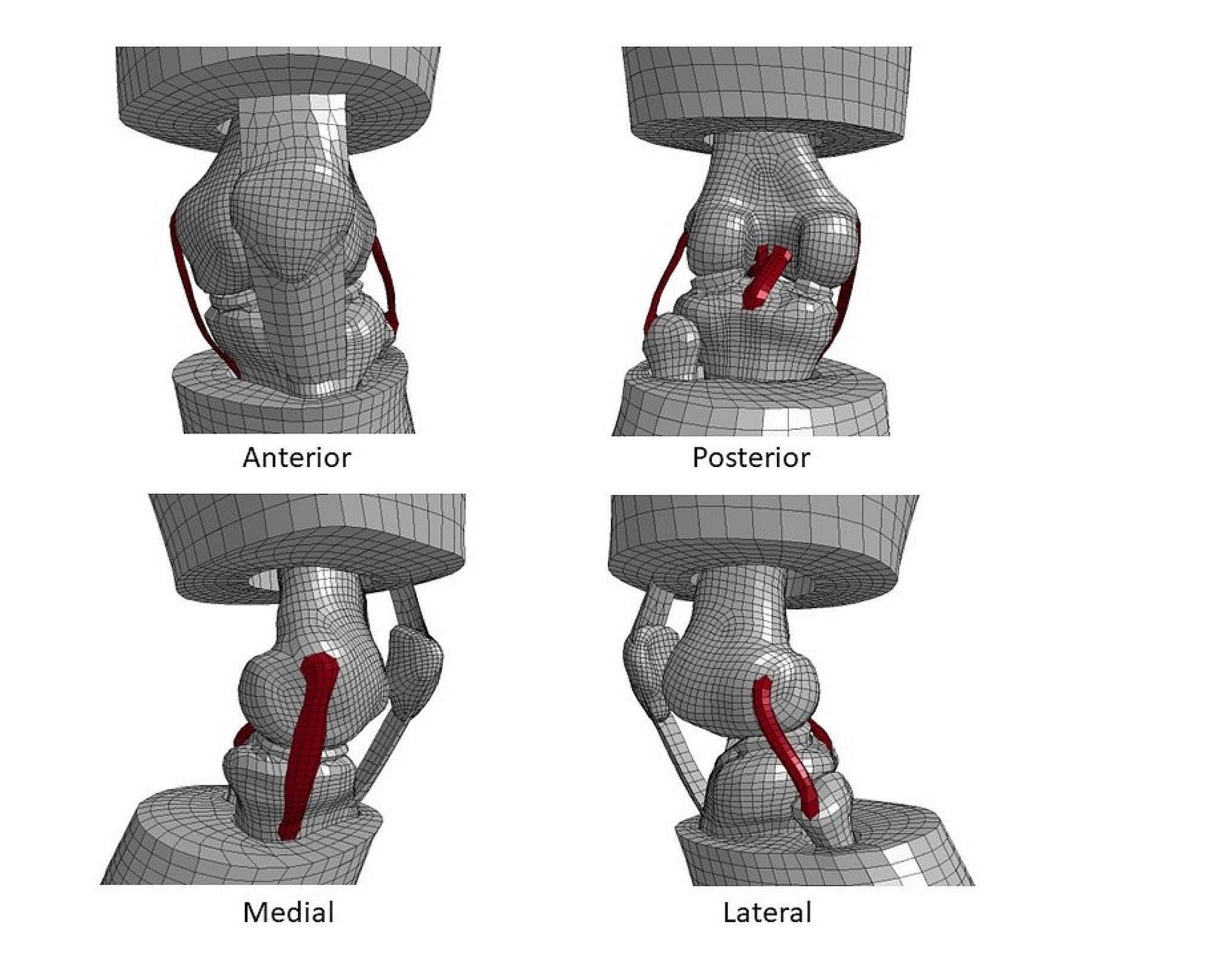
Reference model in undeformed configuration at an inclination angle of 25 degrees. The cruciate ligaments and the collateral ligaments are marked in red color. For improved visualization, the soft tissue in the central region is hidden

**Fig. 2:**
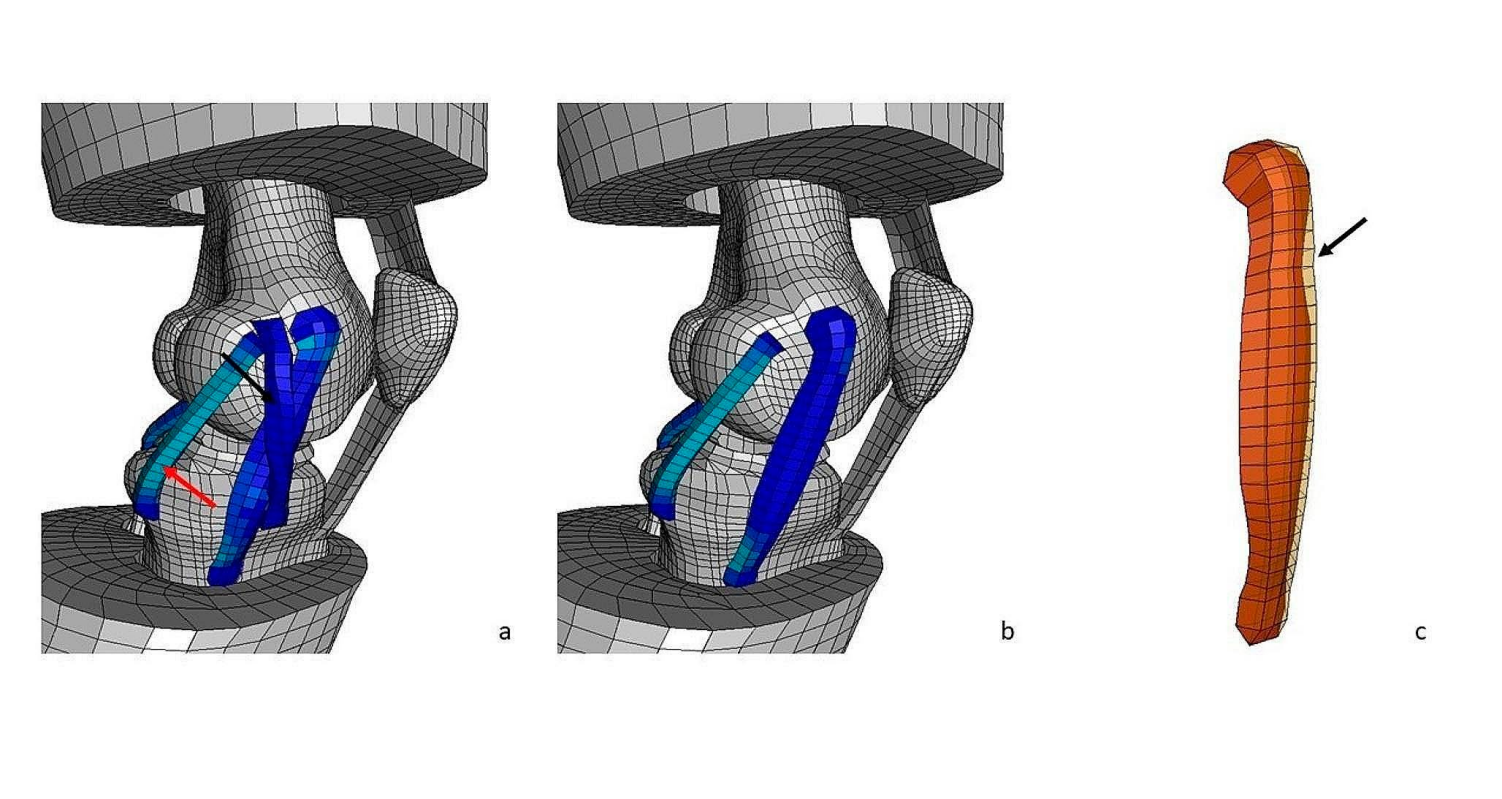
Knee model at the medial side with MCL, attached AML (black arrow) and POL (red arrow) (**a**) and attached augmented sMCL and POL (**b**). Isolated MCL with transparent augmented sMCL (black arrow) (**c**)

The left knee of the GHBMC M50 full human body finite element model with anatomical ACL, posterior cruciate ligament (PCL), lateral collateral ligament (LCL), medial collateral ligament (MCL) and an intact medial and lateral meniscus was isolated (Fig. 1). Five additional anatomic structures (Anteromedial stabilization/anteromedial ligament, *AML*, augmented superficial medial collateral ligament, *sMCL*, posterior oblique ligament, *POL*, anterolateral ligament, *ALL*, and popliteal tendon, *PLT*), were added to the human knee model separately and then all together. The additional extraarticular anteromedial stabilization was applied on the medial side and it was set more oblique compared to the anterior part of the sMCL with a course similar to the ALL on the lateral side and was called “imaginary” anteromedial ligament (AML). The femoral footprint was 4 mm posterior and 8 mm proximal to the medial epicondyle and the tibial footprint at a point of the tibial footprint of the ALL (between the Gerdy tubercle and the fibular head, 10 mm below the medial joint line) but mirrored from the midpoint of the tibia plateau to the medial tibial side (Fig. 2). The length of the AML was measured in flexion and extension and showed no differences. Thus, the femoral and tibial AML fixation was performed isometrically. The length of the AML prior to the rotation was 56.8 mm. For the sake of simplicity, any pre-stresses in the ligaments were neglected. The reference configuration of the model was assumed to be completely stress-free and undeformed.

An external-internal torque was applied to the femur (tibial external rotation) section which is defined rigid with and without the three additional anatomic structures by an external load.

The torque was applied about the longitudinal axis of the femur. The longitudinal femoral axis was defined by the center points of the femur at the femur section and the center point on the tibial plateau. The translational movement of the femur section along the transversal direction was restricted. The translational movement along the longitudinal axis was left free, also the remaining rotational degrees of freedom. The tibia section was defined rigid and completely fixed in space, all translational and rotational degrees of freedom were restricted. All other parts of the model were deformable.

The intensity of the applied torque has been selected by trial and error in order to establish a loading scenario where the ligaments are subjected to an intermediate stress level. The intensity should be strong enough to reveal the relative load distribution in all ligaments of interest, but still far below the failure limit to avoid unphysiological results. The maximum forces carried by the ligaments were in the range of 200–400 N and seem to be realistic. Due to Kennedy et al. [28], the tensile strength of a medial collateral ligament is in the range of 467 ± 33 N.

The normal force histories within the ACL, PCL, LCL and MCL and in each extra-articular structure were measured and the final rotational displacements of the rotated body part were determined for each case. Along the half-length of all ligaments in the model, cross-sections have been defined for tracing the forces carried by the ligaments along their longitudinal directions. The location and the orientation of the cross-sections were updated each cycle according to the current nodal positions.

Data collection and descriptive statistical analysis was performed with Statistical Package for the Social Science (SPSS) after measurements.

## Results

**Fig. 3:**
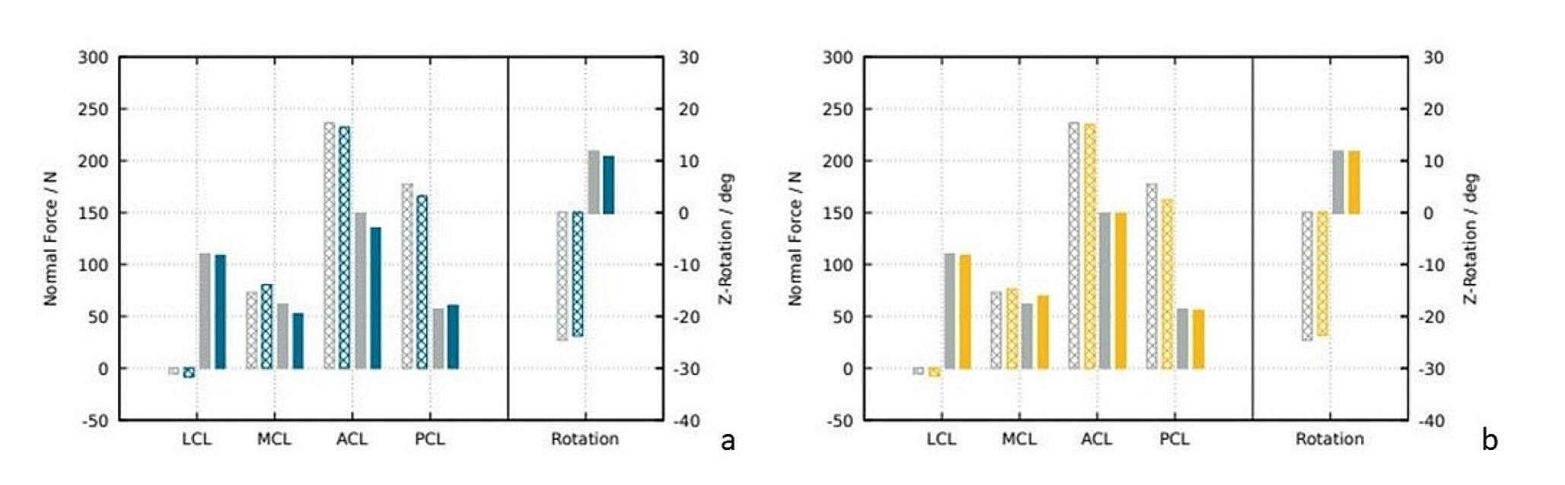
**a**: Force history under internal (dashed) and an external (plain) tibial rotation with attached Anteromedial Ligament (blue) and without (grey). **b**: Force history under internal (dashed) and an external (plain) tibial rotation with attached augmented sMCL (yellow) and without (grey)

Our finite element model showed that the additional anteromedial stabilization procedure or imaginary AML reconstruction was the main secondary stabilizer of tibial external rotation (90% of overall ACL force reduction) and the ALL was the main secondary stabilizer of the tibial internal rotation (50% of overall ACL force absorption). The AML reduced the load on the ACL by 9% in tibial external rotation which could not be achieved by an augmented sMCL (-1%). The AML had no influence in tibial internal rotation (-1%) (Table 1; Fig. 3). In contrast, the ALL reduced the load on the ACL in tibial internal rotation by 21% but had no influence in tibial external rotation (-2%). There was no load reduction on other ligaments (PCL, LCL, MCL) by the ALL and AML (Fig. 3). In the combined measurements with all additional structures (AML, ALL, PLT, POL) the load on the ACL was reduced by 41% in tibial internal rotation. In tibial external rotation the load on the ACL was reduced by 10% with all additional structures (Table 1). An augmented sMCL had only a marginal effect as an additional stabilizer on the ACL in external or internal tibial rotation (Table 1).

**Table 1:** Influence of various extra-articular ligaments on the ACL section force in tibial internal and external rotation. *Ref.*: Reference model, *ALL*: Anterolateral Ligament, *PLT*: Popliteal tendon, *POL*: Posterior oblique ligament, *AML*: Anteromedial Ligament, *sMCL*: augmented superficial Medial collateral ligament

Ref	ALL	PLT	POL	AML	sMCL	Unit	Internal rotation	External rotation
Yes	-	-	-	-	-	N	236,7	149,5
Yes	Yes	-	-	-	-	N	(-21%) 186,0	(-2%) 145,5
Yes	-	Yes	-	-	-	N	(-6%) 220,8	(+ 1%) 152,4
Yes	-	-	Yes	-	-	N	(-8%) 215,7	(-1%) 146,7
Yes	-	-	-	Yes	-	N	(-1%) 232,7	(-9%) 135,3
Yes	-	-	-	-	Yes	N	(-1%) 234,6	(-1%) 148,4
Yes	Yes	Yes	Yes	-	-	N	(-40%) 143,2	(+ 6%) 158,3
Yes	Yes	Yes	Yes	Yes	-	N	(-41%) 138,9	(-10%) 134,0
Yes	Yes	Yes	Yes	-	Yes	N	(-42%) 137,1	(+ 2%) 151,8

## Discussion

This study showed that an additional anteromedial stabilization procedure secures tibial external rotation and has the most protective effect on the ACL during these external rotational forces.

The ALL developed from a newly recognized and small anatomical structure to an important ligament in ACL surgery, especially during revisions [10, 31–33]. Meanwhile many surgeons add an extraarticular anterolateral stabilization procedure in primary or secondary ACL reconstruction to increase graft survival and improve knee stability [7, 10, 34–36]. However, the ALL stabilizes besides anterior tibial translation only internal rotation of the tibia and has no stabilizing function in other mechanism of ACL injury such as valgus stressing or tibial external rotation [15, 37, 38]. Admittedly it is already proven that valgus stressing, and external rotation of the tibia are main mechanisms in ACL injuries, but currently there are no guidelines which considers these facts in treatment regimens for ACL injuries [11, 15, 21, 39, 40]. A distinct structure apart from the ACL which sufficiently stabilizes the tibial external rotation is not evident. At least it is accepted that the medial side of the knee joint plays an important role in ACL injuries or valgus stabilization respectively and its impact is just under intense scientific evaluation [18, 19, 23, 41]. The fact that most of the ACL injuries contain valgus stressing as an injury mechanism, which is mainly stabilized by the MCL, put this structure more and more into the focus [11, 39, 40, 42]. However, surgical treatment algorithms of concomitant MCL lesions in ACL injuries are still lacking [18, 41]. Considering this lack of clarity, the authors of the present study hypothesized that a structure at the medial side of the knee joint very similar to the ALL on the lateral side might play a crucial role in the treatment of combined ACL/MCL injuries. The reason of this hypothesis was that such a structure with such a course could not only stabilize valgus gaping and anterior translation but also tibial external rotation. In the present finite element (FE) knee model study an extraarticular anteromedial stabilization procedure with such a structure with a course similar to the ALL on the lateral side was the most important stabilizer in tibial external rotation of the knee joint. Moreover, it reduced forces on the ACL in tibial external rotation most effectively. Therefore, this additional surgical stabilization of the medial side could be rational in several ACL injuries, especially in injuries which involved valgus stressing or tibial external rotation and with involvement of the MCL and radiological changes as severe lateral bone marrow edema with impression or fluid around the anteromedial capsule and/or the sMCL. The surgical technique of a gracilis tenodesis of Wierer et al. to reconstruct an anteromedial instability and the affected superficial MCL by proximal harvesting of the gracilis tendon and fixation at the medial femoral epicondyle could maybe already considered as an extraarticular anteromedial stabilization procedure or the *Lemaire* reconstruction of the medial knee compartment [25]. It was postulated that this procedure could both restore the MCL stability to bear valgus stressing in slight flexion sufficiently and protect the ACL graft against further stressing. Moreover, recent studies showed that a sMCL graft in combination with an oblique anteromedial stabilization procedure can restore native knee external rotation laxity and achieve better medial knee stability [43, 44]. The present FE knee model study confirmed these assumptions and even showed quantitatively that a procedure like this stabilizes the external tibial rotation and the ACL most effectively. It should be emphasized that this stabilizing effect could not be achieved by an augmentation of the sMCL with or without a reconstruction of the POL. This is also consistent with the existing data about anteromedial stabilization procedures [43, 44]. Finally, it must be mentioned that the AML was responsible for 90% of the overall force reduction on the ACL in tibial external rotation. The ALL for only 50% in tibial internal rotation. This showed the importance of the anteromedial knee structures and the limited compensation mechanisms when these structured are affected. Therefore, the results of present study underlined that combined injuries of the ACL and the MCL should be treated with special care and an additional anteromedial stabilization procedure might be very beneficial in several cases. Even low-grade lesions of the MCL or moderate instabilities after conservative treatment (grad I and II) might be a reasonable indication for surgical stabilization. Whether a common femoral footprint is sufficient and effective for a combined sMCL/AML reconstruction is still a matter of debate. However, future studies are needed to verify these first results of an extraarticular anteromedial stabilization procedure.

The present study has some limitations: First, the human knee model is merely an image of the natural knee and may contain geometric, biomechanical, and material deficiencies so that only biomechanical tendencies may be displayed. Moreover, in present the study the same material model was used for all tested ligaments. This may could have accentuated the results in a quantitative manner. Second, the human knee model includes an intact capsule and an intact medial and lateral meniscus. Therefore, a possible influence of these structures to the load of the ACL or the knee stability in external and internal rotation is not considered. Third, the mechanism of an ACL rupture is mostly a combination of rotational forces and valgus/varus stressing as well as tibial translation. In the present study only the internal and external rotation were considered in 25° flexion. Therefore, structures like the MCL or LCL and the influence of structures in other flexion angles might be underestimated in its function as a protector of the ACL.

## Conclusion

This study showed that an additional anteromedial stabilization procedure secures tibial external rotation and has the most protective effect on the ACL during these external rotational forces.

### Electronic supplementary material

Below is the link to the electronic supplementary material.

Supplementary Material 1
