# Supplementary material for: An anteromedial stabilization procedure has the most protective effect on the anterior cruciate ligament in tibial external rotation. A human knee model study
Source: Arch Orthop Trauma Surg. 2024 May 10;144(6):2703–10. doi: 10.1007/s00402-024-05357-8 (PMC11211157; doi:10.1007/s00402-024-05357-8)
Supplement: Supplementary file 1 — Supplementary Material 1 [file 402_2024_5357_MOESM1_ESM.pdf]

Conflict of Interest and Authorship Conformation Form

Please check the following as appropriate:

x All authors have participated in (a) conception and design, or analysis and interpretation of the data; (b) drafting the article or revising it critically for important intellectual content; and (c) approval of the final version.

x This manuscript has not been submitted to, nor is under review at, another journal or other publishing venue.

x The authors have no affiliation with any organization with a direct or indirect financial interest in the subject matter discussed in the manuscript

x The following authors have affiliations with organizations with direct or indirect financial interest in the subject matter discussed in the manuscript:

Author's name	Affiliation
Fabian Blanke	University Rostock, Germany
Matthias Boljen	Fraunhofer Institute, Germany
Christoph Lutter	University Rostock, Germany
Nicola Oehler	Hessing Stiftung Augsburg, Germany
Thomas Tischer	University Rostock, Germany
Stephan Vogt	Hessing Stiftung Augsburg, Germany
